# IoT Service Clustering for Dynamic Service Matchmaking

**DOI:** 10.3390/s17081727

**Published:** 2017-07-27

**Authors:** Shuai Zhao, Le Yu, Bo Cheng, Junliang Chen

**Affiliations:** 1 State Key Laboratory of Networking and Switching Technology, Beijing University of Posts and Telecommunications, Beijing 100876, China; chengbo@bupt.edu.cn (B.C.); chjl@bupt.edu.cn (J.C.); 2 China Mobile Information Security Center, Beijing 100033, China; yule@chinamobile.com

**Keywords:** Internet of things, semantic similarity measurement, multidimensional model, service clustering

## Abstract

As the adoption of service-oriented paradigms in the IoT (Internet of Things) environment, real-world devices will open their capabilities through service interfaces, which enable other functional entities to interact with them. In an IoT application, it is indispensable to find suitable services for satisfying users’ requirements or replacing the unavailable services. However, from the perspective of performance, it is inappropriate to find desired services from the service repository online directly. Instead, clustering services offline according to their similarity and matchmaking or discovering service online in limited clusters is necessary. This paper proposes a multidimensional model-based approach to measure the similarity between IoT services. Then, density-peaks-based clustering is employed to gather similar services together according to the result of similarity measurement. Based on the service clustering, the algorithms of dynamic service matchmaking, discovery, and replacement will be performed efficiently. Evaluating experiments are conducted to validate the performance of proposed approaches, and the results are promising.

## 1. Introduction

### 1.1. Background

The Internet of things (IoT) integrates user requirement, cyberspace and physical space, which enables the seamless cooperation of human-machine-thing. SOC (Service-Oriented Computing) proposes techniques for provision, selection, discovery, and composition of Web services, and integrates heterogeneous and complicated software entities together organically [1,2]. As the adoption of service-oriented paradigms in the IoT environment [3], real-world devices will open their capabilities through service interfaces, which enable other functional entities to interact with them. In an IoT application, it is indispensable to find suitable services for satisfying users’ requirements or replacing the unavailable services.

With the rapidly growing number of IoT services, discovery and selection for numerous services under the dynamic and large-scale environment of IoT is becoming a crucial task. Several middleware solutions have been proposed for the integration of the physical world with the Web, such as OpenIoT [4], GSN (Global Sensor Networks) [5], and Xively [6]. These solutions act as service platforms that manage millions of services around the world, which enable people to share and monitor environmental data from objects that are connected to the Web. However, most leading middleware solutions provide only limited service discovery and selection functions. It is effective to discover services through service matchmaking techniques [7,8]. However, from the perspective of performance, it is unreasonable to discover services from online repositories directly in the context of the IoT-scale environment [9,10]. Instead, if services are classified offline or clustered according to their similarity, and then the online examining of services will be controlled within several limited clusters. Then, the performance of finding desired services online will be optimistic [11,12]. Besides, because of the dynamic of IoT, the selected services may become unavailable or unfit for the current context, so re-selection and replacing services by similar services from the same cluster is necessary. Therefore, techniques that cluster services offline according to their similarity are critical for dynamic service matchmaking, discovery, and replacement [13].

### 1.2. Motivation

As the research [14] discusses, nearly 12,000 Web services are active on the Web. Even in such conditions, the similarity measurement and clustering of Web service has become a challenging problem. The same issue of IoT service will become a much more complex challenge due to the scale and complexity of IoT. As the IoT service acts on ternary space (i.e., user, cyber, and physical space) rather than Web service that only exists in cyber space, the context of IoT service is more complex than Web service. IoT services imply multidimensional semantic, for instance, the physical quantity observed by the service, the observation capabilities of service, the observation area of the service, and so on. When clustering or measuring similarity between services, these information should be taken into consideration.

A bundle of approaches about measuring similarity between Web services has been proposed in recent years. Basically, it can be divided into: information content-based approaches [15,16] and semantic-network-structure-based approaches [17,18]. However, it is inappropriate that directly using existing approaches on IoT services. Existing similarity measurements focus on the hierarchy and inheritance relations between services in the semantic model. They ignore the relation types, relation contexts, and relation restrictions that imply meaningful semantic information for distinguishing service. Besides, service nodes in a semantic model are defined with data-type and object-type properties. The properties should also not be ignored when computing the similarity between services.

Moreover, existing service models mix multiple feature dimensions of IoT service to construct complicated models. The dimension-mixed model cannot obtain a well-defined taxonomy structure. Thus, using semantic structure-based algorithms to measure the dimension-mixed model will not achieve a satisfactory accuracy. Besides, the property and restriction descriptions of multiple dimensions interfere with each other when measuring similarity based on service description. Therefore, using the existing algorithms to measure IoT services will not conform to the equivalence soundness and disjointedness incompatibility principles of similarity measurement, and the measurement results cannot reflect the real similarity between services [19]. Without accurate similarity measurement, it is impossible to obtain satisfactory clustering results, which will influences the effect of follow-up matchmaking and discovery of services.

This paper proposes the multidimensional semantic model for describing IoT services. Each dimension constructs a semantic model including well-defined service classification, service properties, and property constraints. Based on this multidimensional service model, we propose an MDM (Multiple Dimensional Measuring) algorithm to calculate the similarity between services on each dimension by taking both model structure and model description into consideration. The similarity between services on each dimension is measured concurrently. If the context of service changes, MDM just needs to re-measure the similarity of changed dimensions, rather than existing approaches which require re-measuring the whole similarity. Thus, compared with dimension-mixed approaches, MDM is more accurate and efficient. After that, based on the result of similarity measurement, we employs density-peaks-based clustering [20] to divide services into clusters according to the distribution of their similarity. The similar service clusters are generated automatically without the artificial estimating of parameter (e.g., cluster size or number of cluster). Different services have personalized cluster sizes, which take the heterogeneity of service context into consideration. After clustering, the agile service matchmaking and discovery are possible. In particular, this paper has the following contributions:

1. This paper proposes the MDM algorithm for measuring the similarity between IoT services based on multidimensional service model. The accuracy and efficiency of MDM outperform the dimension-mixed approaches.
2. MDM algorithm employs a density-peaks-based clustering approach to gather similar services together according to the actual distribution of services. It avoids the complicated process of estimating or optimizing parameters.
3. To evaluate the applicability of proposed approaches, we use a combined data set including real and synthetic data. The experiment results indicate that the performance of proposed approaches are applicable to real-life scenarios.

## 2. Preliminaries: Multidimensional Service Model and Model Vectorization

A series of works have been proposed to formally describe IoT services in ontology models, such as references [21,22,23,24,25,26,27,28]. However, existing models mix multiple feature dimensions of IoT service to construct complicated models. Therefore, in a model hierarchy multiple classifying criterions are referenced. The dimension-mixed model cannot obtain a well-defined taxonomy structure, and the distance and positional relationships between nodes are meaningless to reflect the similarity between services. Therefore, using semantic structure-based algorithms to measure the similarity between services will not achieve a satisfactory accuracy. Besides, the restrictions and property descriptions of different dimensions interfere with each other when measuring similarity based on service descriptions.

**Figure 1:**
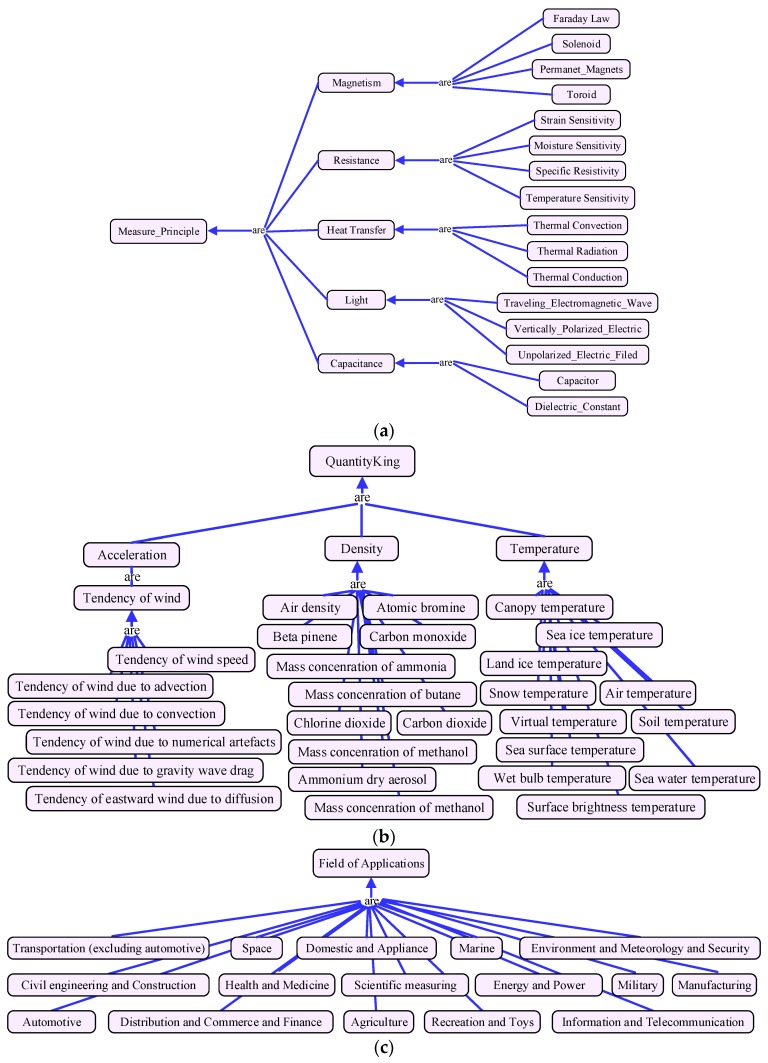

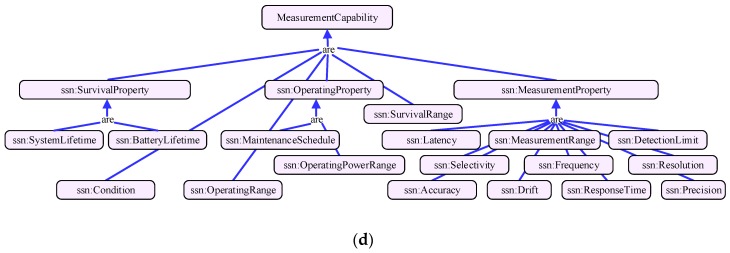
Parts of multidimensional service model. (**a**) Measurement principle dimension; (**b**) Measurement quantity type; (**c**) Application domain dimension; (**d**) Measurement capability dimension

Based on the multidimensional service model proposed in our previous work [29], the service classification, service properties, and property constraints of each dimension are well defined. Then, the MDM algorithm discussed in Section 3 can calculate the similarity between services on each feature dimension accurately and concurrently. To reflect the similarity meeting the perspectives of different users, the whole similarity values are aggregated by users’ personalized weight values. In this section, four representative dimensions are described to demonstrate the idea of multidimensional model, shown in Figure 1. The other parts of service model and the detailed discussion about the problem of existing model are presented in [29].

Figure 1a shows the dimension of observation principle, which is based on the standard definition of observation physical principle described in [30]. As a sensor is a converter of transforming nonelectrical effects into electrical signals, several steps are needed before outputting the electric signal. For example, the measurement principle of capacitive water-level sensor is dielectric-constant. This sensor is fabricated in a form of a coaxial capacitor where the surface of each conductor is coated with an isolating layer. If the water level increases, water occupies more and more space between the coaxial conductors, then transforming the capacitance. The model of this dimension is helpful to discover suitable services according to users’ application scenarios. For instance, magnetic sensors are unfit for the environment with magnetic interference.

Figure 1b depicts the dimension of observation quantity type. It defines the physical quantities that be measured by the IoT services. The quantity type model is a key criterion for service matchmaking; it avoids the ambiguous representation of physical quantity. For instance, the services of body temperature and environment temperature have the similar type of output. Without an exact definition of quantity type, a body temperature service may be offered to user when he requires observing the ambient temperature. The model is constructed based on the Climate and Forecast standard of W3C CF (Climate and Forecast)-feature ontology [31], which makes a standard definitions for common observed physical quantity. For instance, it has more than 50 quantity types to express temperature, such as surface air temperature, canopy temperature, and dry-bulb temperature, etc.

Figure 1c indicates the dimension of application domain deriving from reference [30]. This dimension will help users to choose the services that are fit for their application domain. For instance, if we select a service to measure the gas concentration in coalmine domain, the service must be coalmine dedicated and “intrinsically-safe”.

Figure 1d shows the measurement capability dimension. This model is derived from the capability model of W3C SSN (Semantic Sensor Network) ontology [32]. Due to the performance of IoT service may be influenced by operation environment, this model expresses the measurement capabilities of services under certain conditions, consisting the concepts of sensitivity, frequency, drift, and accuracy, etc. It can be used to check whether the service has been properly used or to determine how a service will perform in a particular environment. It is also an important criterion for service matchmaking. For instance, the capability of a temperature observation service is: with temperature −200 to 500 °C the accuracy is ±1.0 °C, while from 500 to 800 °C it is ±0.5%.

Based on the formally multidimensional model, the semantic similarity between IoT services can be measured. Before measuring, the model of services should be vectorized, that is, transforming the model description of a service to a tuple of terms. The model conversion approach of [33] is adopted. After vectorization, the semantic concept C will be denoted as a tuple, as Equation (1) defines:

(1) $$tuple=\;\left\{C,\;\left[\delta_{i},\;\gamma_{\delta_{i}}\right]\;,\;\left[o_{j},\gamma_{o_{j}}\right],\;\left[o_{j},\;C_{o_{j}}^{x}\right],\;C_{o_{j}}^{x},\lambda_{o_{j}}^{y}\right\}$$

where in OWL-annotated semantic documents, C is the name (or URI) of the concept C, each [] is a property term including a property and its restriction, $\delta_{i}$ (i=1…n) is a datatype property of the concept $C,\gamma_{\delta_{i}}$ is a restriction for the datatype property $\delta_{i}$, $o_{j}$(j=1…m) is an object property of the concept C, $\gamma_{o_{j}}$ is a restriction for the object property $o_{j}$, $C_{o_{j}}^{x}$(x=1…k) is a concept related by the object property $o_{j}$, and $\lambda_{o_{j}}^{y}$ is a Boolean operation between concepts $C_{o_{j}}^{x}$.

After the model vectorization, the semantic description of an IoT service i can be represented as a tuple:

$$tuple_{i}=\left\{term_{1},term_{2},\ldots ,term_{i},\ldots ,term_{n}\right\}$$

**Figure 2:**
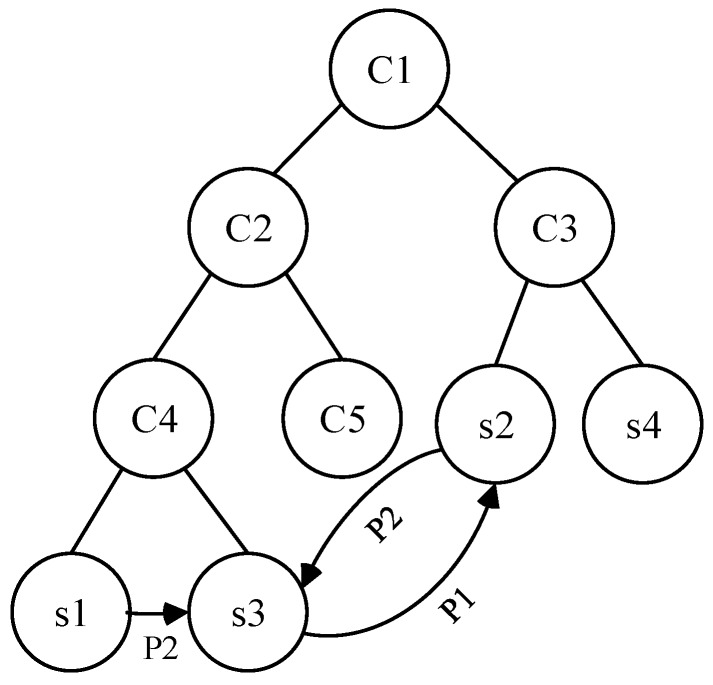
An example of service vectorization.

**Example** **1.** *We use a simplified model structure (shown in Figure 2) to demonstrate the process of model vectorization. In this model,* C1  *to*  C5 *are classes that form the inheritance structure,* s1 *to* s4 *are service instances (i.e., objects that belong to different classes).* P1 *and* P2 *are object properties that denote the relationships between service instances. Assuming that we want to measure the similarity between* s1*,*  s2*, and* s3*. Before similarity measurement, we should vectorize the model of services into tuples as following according to above discussion:*

$$tuple_{s1}=\left\{s1,s3,C4,P2,typeOf,\left[typeOf,C4\right],\left[P2,s3\right]\right\}\;;$$

$$tuple_{s2}=\left\{s2,s3,C3,P2,typeOf,\left[typeOf,C3\right],\left[P2,s3\right]\right\}\;;$$

$$tuple_{s3}=\left\{s2,s3,C4,P1,typeOf,\left[typeOf,C4\right],\left[P1,s2\right]\right\}\;;$$

## 3. MDM Similarity Measurement

Before clustering IoT services, the similarities (or distances) between services should be measured based on MDM. MDM matches both the structure information of the model hierarchy and the description of service properties, relations and restrictions. It employs Li’s approach [34] as the similarity computing method of structure information, which proposed a hybrid semantic similarity model by adopting a nonlinear model. For measuring the similarity of service description, based on the model vectorization algorithm discussed in Section 2, it adapts the *TF-IDF* (Term Frequency and Inverse Document Frequency) [35] and Cosine Similarity to calculate the similarity of service tuples. By combining the similarity of structure and description, MDM can measure the similarity of every dimension accurately and concurrently. Then, the overall similarity will be generated by aggregating the similarity of multiple dimensions according to users’ preferences, for instance, allocating different weights for different dimensions.

### 3.1. Structure Similarity

A series of algorithms for measuring structure similarity have been proposed, considering the aspect of information content [15,16], depth in the hierarchy [36,37], semantic density [34,38], and shortest path length [39,40], etc. In order to achieve a good similarity measure, Li [34] investigated the effectiveness of a variety of strategies considering possible structure information. Its research results demonstrate that comparing the performance against human common sense is the only way to evaluate the quality of a method for calculating concept similarity. Therefore, the closer the result compares with human judgment, the better it will be. The work of [34] has confirmed the hypothesis that the human judgment of similarity is a nonlinear process. Its measurement algorithm, which models the length and depth of shortest path into a nonlinear function and combines them by multiplication, can obtain a dramatic improvement compared to previous methods. We employ their approach to calculate the structure similarity of services. Given the service a and b, the structure similarity on dimension i between their class C can be measured by Equation (2).

(2) $$Sim_{C_{i}}\left(a,b\right)=\{\begin{array}{c} e^{-\alpha l}\cdot \frac{e^{\beta h}-e^{-\beta h}}{e^{\beta h}+e^{-\beta h}}\;if\;C_{ai}\neq C_{bi}\\ 1\;if\;C_{ai}=C_{bi} \end{array}$$

where h denotes the depth of the subsume Class of $C_{ai}$ and $C_{bi}$, and l is the shortest path length between $C_{ai}$ and $C_{bi}$. α and β are the impacts of l and h. Li [34] configures the optimal parameters that α = 0.2 and β = 0.6. Under these parameters, the correlation coefficient between this measurement and human similarity judgments is 0.8914, while correlation between different people is 0.9015. It indicates that the measurement performs nearly at a level of human replication.

### 3.2. Service Description Similarity

Assuming that S is the candidate service set $S=\left\{s_{1},s_{2},\ldots ,s_{i},\;\ldots ,s_{m}\right\}$, then according to Equation (1), S can be represented as:

$$S=\left\{tuple_{1},tuple_{2},\ldots ,tuple_{i},\;\ldots ,tuple_{m}\right\}$$

Then we construct the feature vector of each service using the *TF-IDF*. *TF-IDF* is the product of two statistics: term frequency (*TF*) and inverse document frequency (*IDF*). The former is the frequency of a term in a document, while the latter represents the occurrence frequency of the term across all documents. It is obtained by dividing the total number of documents by the number of documents containing the term and then taking the logarithm of that quotient. The higher *TF-IDF* of a term, the more important it is for a document. In our study, corpus is the service set, document and term are tuple and description term respectively. We adopt *TF-IDF* to calculate the frequency of terms in the service tuple. The *TF* of a term in a service tuple is:

$$TF_{term}=\frac{f}{\left|tuple\right|}$$

|tuple| is the size of terms of the tuple, and f is the occurrence frequency of term in this tuple. The IDF of the term can be measured by:

$$IDF_{term}=log\frac{\left|S\right|}{\left|\left\{tuple\in S:term\in tuple\right\}\right|}$$

The cardinality of service set S is denoted as |S|, and |{tuple∈S:term∈tuple}| represents the amount of tuples that includes the term. Thus, the TF−IDF can be calculated by:

$$TF-IDF_{term}=TF_{term}\cdot IDF_{term}$$

Then, a vector of a service by calculating the *TF-IDF* of terms in its tuple is obtained. For a service s, its tuple $tuple_{s}=\left\{term_{1},term_{2},\ldots ,term_{i},\ldots ,term_{k}\right\}$ and its vector is:

$$vector_{s}=\left\{TFIDF_{1},TFIDF_{2},\ldots ,TFIDF_{i},\ldots ,TFIDF_{k}\right\}$$

The similarity between two vectors can be measured by the cosine-similarity. The *IDF* not only strengthens the effect of terms whose frequencies are very low in a tuple, but also weakens the effect frequent terms. For instance, the property subClassof: Thing occurs in most ontology concepts, then the IDF of it is close to zero. Therefore, the terms with low IDF value will have weak impact on the cosine similarity measurement. The description similarity on the dimension d between two services i and j can be measured by:

(3) $$Sim_{P_{d}}\left(i,j\right)=\frac{vector_{i}\cdot vector_{j}}{\left|\left|vector_{i}\left|\left|\cdot \right|\right|vector_{j}\right|\right|}\;$$

### 3.3. Multidimensional Aggregation

The similarity in the i dimension between two services a and b can be calculated by combining $sim_{C}$ (Equation (2)) and $sim_{P}$ (Equation (3)). δ is the impact parameter which indicates the effect of structure information on the similarity measurement.

(4) $$Sim_{i}\left(a,b\right)=\delta \cdot sim_{C_{i}}\left(a,b\right)+\left(1-\delta \right)\cdot sim_{P_{i}}\left(a,b\right)$$

The similarity values of each dimension can be aggregated by weights according to the users’ preferences:

(5) $$Sim\left(a,b\right)=\overset{n}{\underset{i=1}{\sum }}w_{i}\cdot sim_{i}\left(a,b\right)\;,\;\overset{n}{\underset{i=1}{\sum }}w_{i}=1\;$$

where n is the dimension number of semantic service model.

## 4. IoT Service Clustering

This paper employs density-peaks-based clustering [20] to divide services into clusters according to the potential density distribution of similarity between services. Density-peaks-based clustering is a fast and accurate clustering approach for large-scale data. After clustering, the similar services are generated automatically without the artificial determining of parameter. The distance between two services can be calculated by Equation (6):

(6) Dist(a,b)=1−Sim(a,b)

### 4.1. Local Density and Distance Calculating

The density-peaks algorithm is based on the assumptions that cluster centers are surrounded by neighbors with lower local density, and they are keep a large distance from other points with higher density. Assuming that $S={\left\{s_{i}\right\}}_{i=1}^{N}$ is the service set that will be clustered, $s_{i}$ is a service of S, $I_{S}=\left\{1,2,\ldots ,N\right\}$ is the set of index. For each service $s_{i}$ in S, two quantities are defined: its local density $\rho_{s}$ and its distance $\theta_{s}$ from services of higher density. The local density ρ of service i is defined as:

(7) $$\rho_{i}=\underset{j}{\sum }\chi \left(d_{ij}-d_{c}\right)$$

where $d_{c}$ is a cutoff distance. If x<0, χ(x) equals to 1, otherwise χ(x)=0. θ is calculated by measuring the closest distance between the service i and other services with higher density than i:

(8) $$\theta_{i}=\underset{j:\rho_{j}>\rho_{i}}{\min}d_{ij}$$

For the service with highest density, its density is defined as: $\theta_{i}=max_{j}\left(d_{ij}\right)$. Note that $\theta_{i}$ is much larger than the typical nearest neighbor distance only for services that are local or global maxima in the density. Algorithm 1 describes the procedure of calculating clustering distance. Firstly, the data density are sorted in descending order, set ${\left\{q_{i}\right\}}_{i=1}^{N}$ is the index generated from the descending order, i is the index of descending order and $q_{i}$ is the original index. Then, the clustering distance $\theta_{i}$ of service $s_{i}$ is calculated by $\theta_{i}=Dist\left(i,j\right)$, $s_{j}$ is the service that has larger density (than $s_{i}$) and closest to $s_{i}$. In S, we use $n_{i}$ to denote the index of $s_{j}$, namely, $n_{i}=j$. ${\left\{n_{i}\right\}}_{i=1}^{N}$ is defined as:

(9) $$n_{q_{i}}=\{\begin{array}{c} arg\underset{\begin{array}{c} q_{j}\\ j<i \end{array}}{\min}\left\{d_{q_{i}q_{j}}\right\},\;i\geq 2;\\ 0,\;i=1. \end{array}$$

The clustering distance of the point with largest density is defined as $max_{j}\left(d_{ij}\right)$ that is the maximum value of all data points, and the index is $n_{q_{1}}=0$.

**Algorithm 1.** Calculating Clustering Distance**Input:**F: the matrix of distances between services;
ρ: local density of each service;**Output:**θ: the clustering distance of each service;
$n_{q_{i}}$: the index of service that has larger density and closest to $s_{q_{i}}$;**Sort in descending order by density**
ρ
1: ${\left\{\rho_{i}\right\}}_{i=1}^{N}$ 2: $q_{i}$←descending order index of density; **Distance assignment of**
θ 3: ${\left\{n_{i}\right\}}_{i=1}^{N}$← 0; 4: **for** i:=1 to N **do** 5: $\theta_{qi}$←$d_{max}$; 6:   **for** j:=1 to i−1 **do**
7:     **if**
$dist\left(s_{q_{i}},s_{q_{j}}\right)<\;\theta_{qi}$
**then** 8:       $\theta_{qi}=dist\left(s_{q_{i}},s_{q_{j}}\right);$ 9:       $n_{q_{i}}=q_{j}$; 10:     **end if** 11:   **end for** 12: **end for** 13: $\theta_{q_{1}}=\underset{j\geq 2}{\max}\;\theta_{j};$


### 4.2. Cluster Center Selecting

For services ${\left\{x_{i}\right\}}_{i=1}^{N}$ in S, their local density and clustering distance can be calculated: ${\left\{\left(\rho_{i},\theta_{i}\right)\right\}}_{i=1}^{N}$. Cluster centers are the services that have both large ρ and large θ. In order to eliminate the difference of magnitude, the ρ and θ of each service are normalized to [0,1]. Then, the values that are comprehensive consideration of ρ and θ are calculated:

(10) $$\gamma_{i}=\rho_{i}\theta_{i},\;i\in I_{S}$$

**Figure 3:**
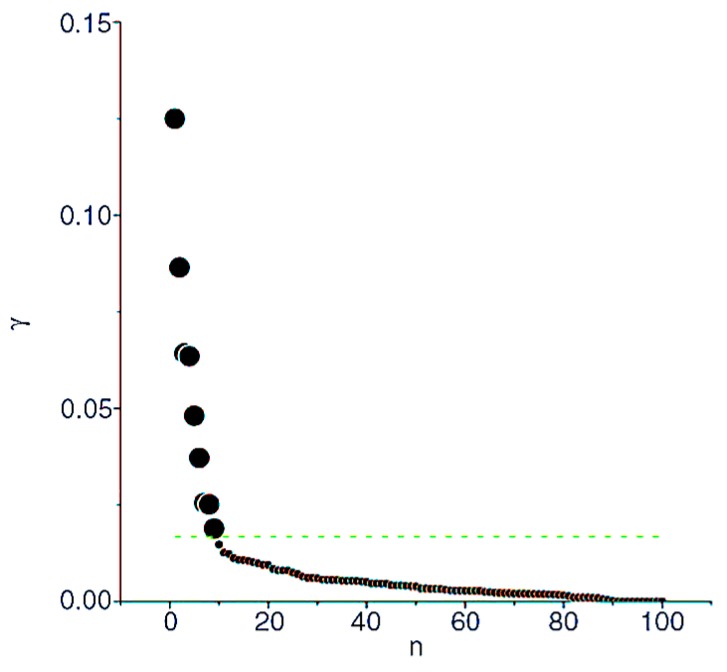
Decision graph for assuming cluster centers. $\gamma_{i}=\rho_{i}\theta_{i}$ is the combination of local density $\rho_{i}$ and clustering distance $\theta_{i}$ of service i. n is the index of services after they are sorted in descending order by γ.

Obviously, the higher value of γ, the more likely it becomes a cluster center. ${\left\{\gamma_{i}\right\}}_{i=1}^{N}$ are sorted in descending order. The sorted γs are drawn on the coordinate plane, the horizontal axis is the index of γ, the vertical axis is the value of γ, as shown in Figure 3. This coordinate plane is defined as decision graph. In addition, then a number of service points are intercepted from front to back as the cluster centers. The decision graph shows that the γ values of cluster centers are larger and discrete, while non-center services are continuous and smooth. The transition of γ value from the cluster centers to the non-center services has a significant “jump”, this “jump” can be detected by numerical detection method [41]. Therefore, the cluster center of the dataset S will be determined according to decision graph and numerical detection method. 

### 4.3. Cluster Assignment

After the center of every cluster is assumed, the next step is to assign non-center services to clusters. Algorithm 2 describes the procedure of cluster assignment. Each service are assigned in the order of density descending, which is from the cluster center services to the cluster core services to the cluster halo services in the way of layer by layer.

Suppose that $n_{c}$ is the total number of cluster centers, naturally, the number of clusters is also $n_{c}$. ${\left\{m_{j}\right\}}_{j=1}^{n_{c}}$ is the index of corresponding service for each cluster center, i.e., service $s_{m_{j}}$ is the center of the jth cluster. ${\left\{c_{i}\right\}}_{i=1}^{N}$ is the cluster of each service belongs to, i.e., service $s_{i}$ belongs to cluster $c_{i}$. According to the definition of ${\left\{n_{i}\right\}}_{i=1}^{N}$ in Equation (9), $n_{i}$ is the index of service which has larger density than ith service ($s_{i}$) and closest to $s_{i}$. 

**Algorithm 2.** Cluster Assignment**Input:**$q_{i}$: the descending order of index according to density ρ;
${\left\{m_{j}\right\}}_{j=1}^{n_{c}}$: the index of cluster center of cluster j;
$n_{c}$: total number of clusters (centers);
$n_{q_{i}}$: the index of service which has larger density than $s_{q_{i}}$ and closest to $s_{q_{i}}$;**Output:**${\left\{c_{i}\right\}}_{i=1}^{N}$: the cluster of each service belongs to, i.e., $s_{i}$ belongs to $c_{i}$;1: ${\left\{c_{i}\right\}}_{i=1}^{N}\leftarrow -1$; //initialization of $c_{i}$ 2: **for** j:=1 to $n_{c}$
**do** 3: $c_{m_{j}}$=j;     //cluster centers 4: **end for** **Non**-**center services assignment** 5: **for** i:=1 to N **do** //descending order of index 6:   **if** $c_{q_{i}}=-1$
**then** 7:     $c_{q_{i}}=c_{n_{q_{i}}}$; 8:   **end if**
9: **end for**


If the dataset has more than one cluster, each cluster can be furthermore divided into two parts: cluster core and cluster halo. The cluster core with higher density is the core part of a cluster. The cluster halo with lower density is the edge part of a cluster. The procedure of determining cluster core and cluster halo is described in Algorithm 3. We define the border region of a cluster as: the border region of cluster $c_{1}$ is consisted by the services $s_{i}$ that belongs to $c_{1}$, and the distance between $s_{i}$ and $s_{j}$ (which belongs to another cluster $c_{2}$) is less than $d_{c}$. An average density bound is defined as ${\left\{\rho_{c_{i}}^{b}\right\}}_{i=1}^{n_{c}}$, $\rho_{c_{i}}^{b}$ is the average density bound of cluster $c_{i}$. If the density ρ of service s is larger than $\rho_{c_{i}}^{b}$, then service s belongs to the core part of cluster $c_{i}$; otherwise, it belongs to the halo part of cluster $c_{i}$.

**Algorithm 3.** Determining Cluster Core and Cluster Halo**Input:**F: the matrix of distances between services;
$d_{c}$: cut-off distance;
${\left\{c_{i}\right\}}_{i=1}^{n_{c}}$: the cluster of each service belongs;**Output:**${\left\{h_{i}\right\}}_{i=1}^{N}$: the signal of core or halo that service $s_{i}$ belongs to;**Initialization** 1: ${\left\{h_{i}\right\}}_{i=1}^{N}$ ← 0; 2: ${\left\{\rho_{c_{i}}^{b}\right\}}_{i=1}^{n_{c}}$ ← 0; 3: **for** i:=1 to N−1 **do** 4: **for** j:=I + 1 to N **do** 5:   **if** $c_{i}\neq c_{j}$ and $dist\left(s_{i},s_{j}\right)<d_{c}$**then** 6:     ${\bar{\rho }}_{ij}=\frac{1}{2}\left(\rho_{i}+\rho_{j}\right)$; 7:     **if**
${\bar{\rho }}_{ij}>\rho_{c_{i}}^{b}$
**then** 8:     $\rho_{c_{i}}^{b}={\bar{\rho }}_{ij}$; 9:   **end if**  10:   **if**
${\bar{\rho }}_{ij}>\rho_{c_{j}}^{b}$
**then** 11:     $\rho_{c_{j}}^{b}={\bar{\rho }}_{ij}$; 12:   **end if**
13: **end if** 14: **end for** 15: **end for** 16: **for** i:=1 to N 17: **if**
$\rho_{i}<\rho_{c_{i}}^{b}$
**then** 18:     $h_{i}=1$;   //belongs to halo part of cluster $c_{i}$ 19:   **end if** 20: **end for**

After clustering, the similar service neighbors are generated automatically without the estimation of parameters. Moreover, different services have personalized neighbor sizes according to the actual density distribution, which may avoid the inaccurate matchmaking caused by constant neighbor size.

## 5. Experimental Evaluation

In this section, we evaluate the performance of proposed MDM measurement and service clustering. We use a combined data set including real and synthetic data, which collects service from multiple sources and adds essential service instances and descriptions. The data sources of combined service set are shown in Table 1. In this paper, 510 real sensor services are collected from 6 sensor sets, including indoor and outdoor sensors. Then, the amount of service is expanded to 1000, and essential semantic service descriptions are supplemented for similarity measuring. The experimental evaluation is performed under the environment of 64-bit Windows 7 Professional, Java 7, Intel Xeon Processor E5-2650 2.3GHz processor, and 32 GB RAM. Section 5.1 discusses about the performance of MDM, and Section 5.2 discusses about the performance of service clustering.

### 5.1. Performance of Similarity Measurement

To evaluate the performance of similarity measurement, we employ the most widely used performance metrics from the information retrieval field. The performance metrics in this experiment are defined as follows:

***Precision***. Precision is used to measure the preciseness of a search system. Precision for a single service refers to the proportion of matched and logically similar services in all services matched to this service, which can be represented by the following equation:

$$Precision=\frac{\left|A\cap B\right|}{\left|B\right|}$$

where *A* is the number of logically similar service and *B* is the number of matched services calculated by MDM.

***Recall***. Recall is used to measure the effectiveness of a search system. Recall for a single service is the proportion of matched and logically similar services in all services that are logically similar to this service, which can be represented by the following equation:

$$Recall=\frac{\left|A\cap B\right|}{\left|A\right|}$$

***F-measure.*** F-measure is employed as an aggregated performance scale for a search system. In this experiment, F-measure is the mean of precision and recall, which can be represented as:

$$F-measure=\frac{2\times Precision\times Recall}{Precision+Recall}$$

When the F-measure value reaches the highest level, it means that the aggregated value between precision and recall reaches the highest level at the same time.

In order to filter out the dissimilar services with lower similarity values, an optimal threshold value is needed to be estimated. In addition, the aggregative metric of F-measure is used as the primary benchmark for estimating the optimal threshold value. Besides, parameter *δ* is the impact of description and structure similarity for similarity measuring. To obtain the best performance, an optimal δ value should also be estimated. The initial values of two parameters are set to 0, and increasing incrementally by 0.1 until 1.0.

**Figure 4:**
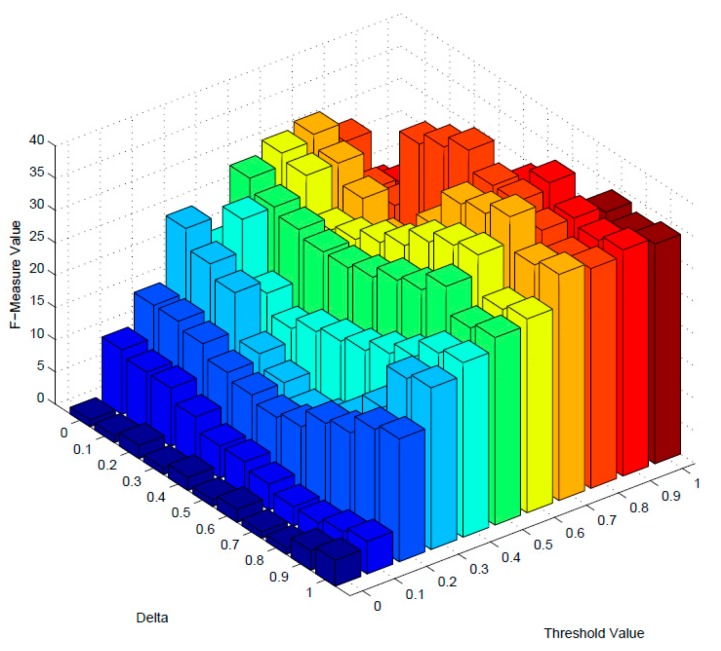
The F-measure of dimension-mixed.

**Figure 5:**
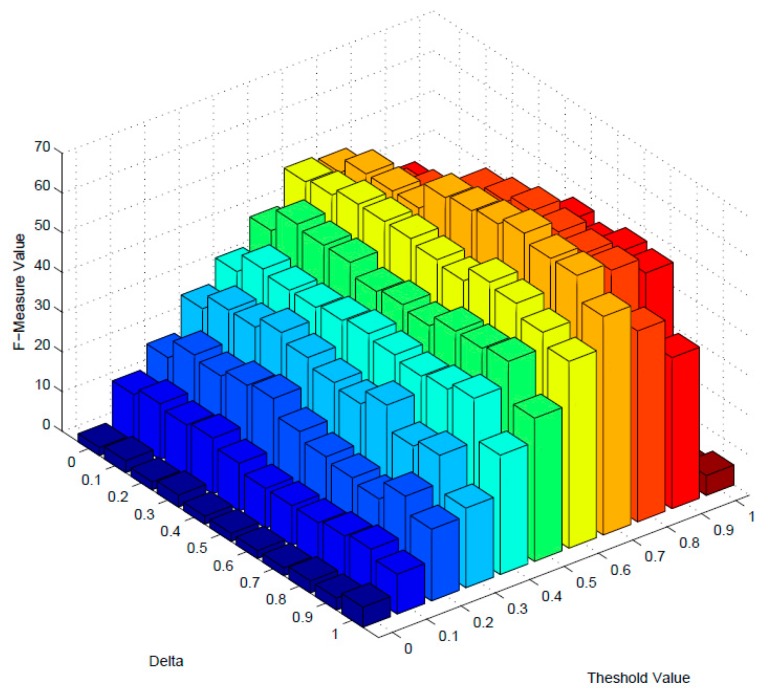
The F-measure of multi-dimension.

Figure 4 and Figure 5 demonstrate the variation of F-measure values of dimension-mixed and multidimensional model as the changing of these two parameters. When the value of F-measure reaches the highest point, it achieves the best performance, and the optimal value of threshold and δ will be determined. As Figure 4 and Figure 5 indicates, *δ* = 0.5 and threshold = 0.8 are the optimal values of dimension-mixed model, and the F-measure is 40 with these parameters; meanwhile *δ* = 0.7 and threshold = 0.7 are the optimal values of multidimensional model, and the F-measure is 63 with these parameters. Besides, the overall F-measure values of multidimensional model are higher than dimension-mixed model.

**Figure 6:**
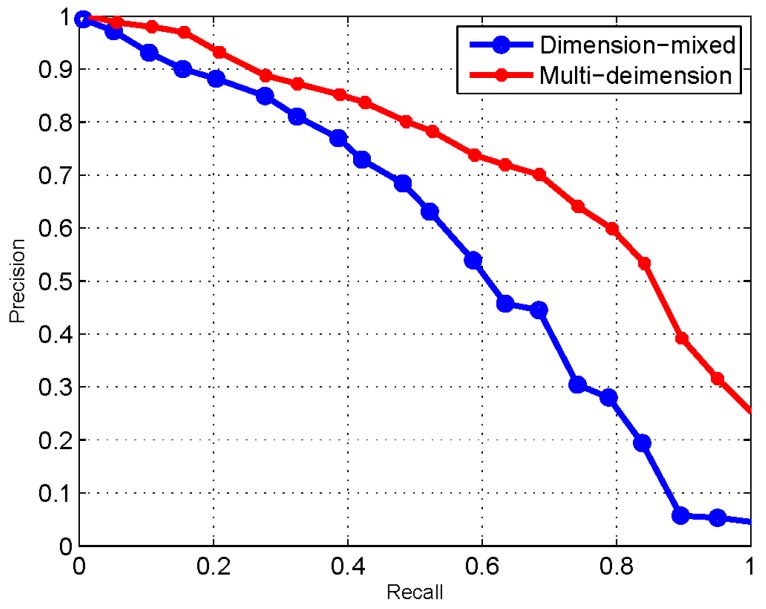
The performance of dimension-mixed and multidimensional.

The performance comparison between multidimensional and dimension-mixed model is shown in Figure 6. As the results indicate, the performance of similarity measurement based on the multidimensional model outperforms to the dimension-mixed way. The reason is that, employing the multidimensional model, both description similarity and structure similarity can be measured accurately. For the structure similarity, each dimension has a well-defined semantic structure in which the distance and positional relationships between nodes are meaningful to reflect the similarity between services. For the description similarity, each dimension only focuses on the descriptions that are contributed to expressing the features of current dimension. Conversely, using the dimension-mixed way, which mixes the semantic structures and descriptions of all dimensions into a complicated model, the measurement can only obtain an overall similarity value.

### 5.2. Performance of Service Clustering

**Figure 7:**
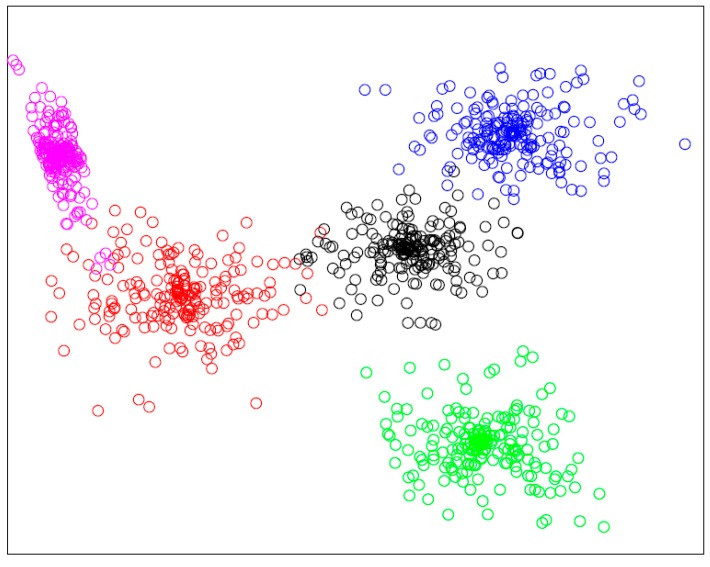
The result of clustering.

In this section, we evaluate the performance of clustering. The number of service that will be clustered is 1000 with essential semantic description and structure, as Table 1 describes. The cut-off distance $d_{c}$ for calculating local density of services is set to 0.03. As Figure 7 shows, although the service set is high overlap in data distribution, the proposed approach successfully detects the cluster structure. The services are clustered into 5 clusters, the borders of clusters are clear, and each cluster is dense and compact.

**Figure 8:**
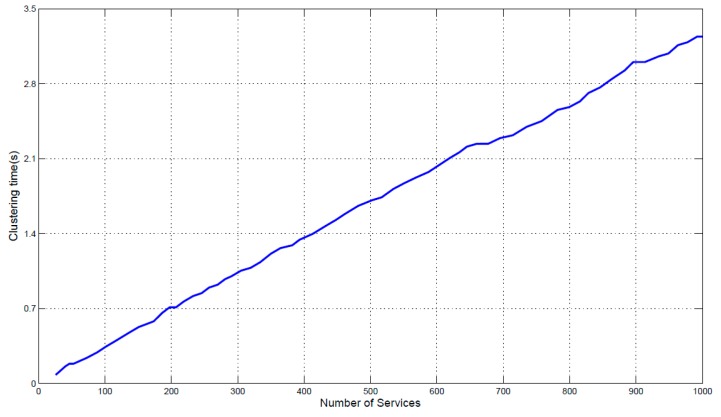
The time of clustering influenced by services number.

**Figure 9:**
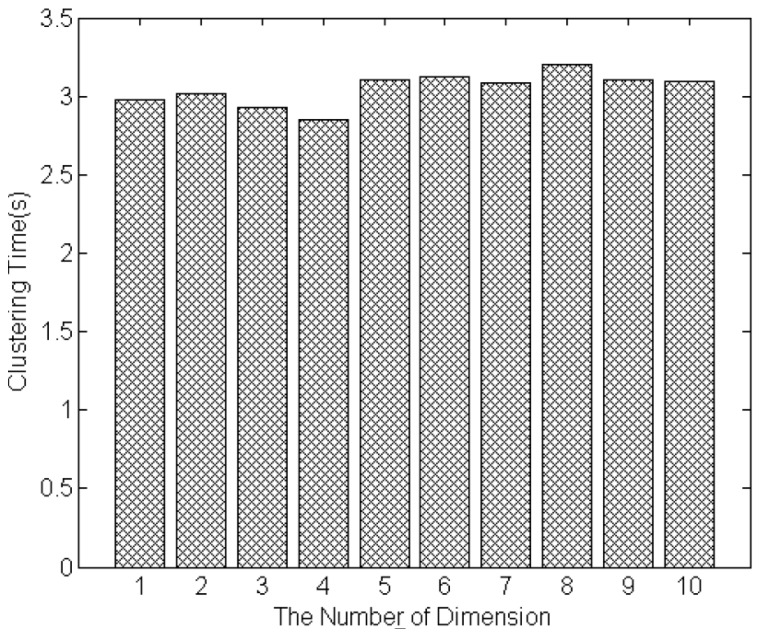
The time of clustering influenced by dimension number.

The size of service set and the number of feature dimensions are two important factors to evaluate the efficiency of proposed clustering approach. Figure 8 shows the time of clustering as the size of services is increased from 100 to 1000. The time of clustering 1000 services is 3.2 s. The results show that the clustering time is linear with respect to the number of IoT services to be clustered, and the clustering time of hundreds services is controlled within a few seconds. Figure 9 shows the time of clustering as the dimensions of service model increasing from 1 to 10. The number of services that will be clustered is set to 1000. The minimum clustering time is 2.8 s, when there are four feature dimensions of the model; and the maximum clustering time is 3.3 s, when the number of feature dimensions is eight. The results show that the clustering time will not increase as the increase of dimension number. It is because that MDM measures each dimension’s similarity concurrently. Thus, the whole time of measuring similarity of all dimensions is equal to the time of single dimension that takes longer time than other dimensions. Besides, the clustering is based on the measurement result of MDM (distances between services), it will not be influenced by the dimension number. Therefore, the proposed approaches improve the accuracy of similarity measurement and service clustering in the condition of not increasing the computation time.

The experimental results demonstrate that, the proposed clustering approach is able to cluster hundreds of IoT services in a reasonable amount of time. In the application domains of IoT SOC paradigm, the number of services usually does not exceed several thousands. Besides, if the scale of services is very large, the service clustering can be performed offline. Thus, the performance of proposed clustering approach is competent for applying in real application scenarios.

## 6. Conclusions

This paper proposes a multidimensional model-based approach to measure the similarity between IoT services. Then, density-peaks-based clustering is employed to gather similar services together according to the result of similarity measurement. A combined data set is used to evaluate the proposed approaches, which collects service from multiple sources and adds essential service instances and descriptions. The experiment results demonstrate that the performance of proposed approaches are promising and applicable to real-life scenarios.

Currently, the experiments are conducted using a centralized single dataset, and the size of test set is limited. Our future works include extending the experiments using distributed datasets and expanding the number of service set. Moreover, we plan to propose a quantitative model to diagnose the quality of service clustering, then to determine when the clustering structure becomes unacceptable and require re-clustering as the evolution of services.

## Figures and Tables

**Table 1:** Service set.

	Source	Number of Services	Number after Expansion
Outdoor	ABM	105	200
	LSM	100	200
	CCMWS	93	200
	DHCIS	76	200
Indoor	IntelLab	54	100
	MavHome	82	100
Total		510	1000
